# Virtual reality-based therapy improves balance and reduces fear of falling in patients with multiple sclerosis. a systematic review and meta-analysis of randomized controlled trials

**DOI:** 10.1186/s12984-023-01174-z

**Published:** 2023-04-11

**Authors:** Irene Cortés-Pérez, María Catalina Osuna-Pérez, Desirée Montoro-Cárdenas, Rafael Lomas-Vega, Esteban Obrero-Gaitán, Francisco Antonio Nieto-Escamez

**Affiliations:** 1 Department of Health Sciences, University of Jaén, Campus Las Lagunillas, s/n, Jaén, Spain; 2 FREMAP, Mutual Collaborator With Social Security Nº 61, Santo Reino, 7, Jaén, Spain; 3 Center for Neuropsychological Assessment and Neurorehabilitation (CERNEP), University of Almería, Almería, Spain; 4 Department of Psychology, University of Almería, Ctra. Sacramento, s/n, La Cañada, Almería, Spain

**Keywords:** Multiple sclerosis, Virtual reality, Postural balance, Postural control, Fear of falling, Gait speed

## Abstract

**Objective:**

This study aims to conduct a meta-analysis to assess the effect of virtual reality-based therapy (VRBT) on balance dimensions and fear of falling in patients with multiple sclerosis (PwMS). Secondarily, to determine the most recommendable dose of VRBT to improve balance.

**Methods:**

PubMed Medline, Web of Science, Scopus, CINAHL and PEDro were screened, without publication date restrictions, until September 30th, 2021. Randomized controlled trials (RCTs) comparing the effectiveness of VRBT against other interventions in PwMS were included. Functional and dynamic balance, confidence of balance, postural control in posturography, fear of falling and gait speed were the variables assessed. A meta-analysis was performed by pooling the Cohen's standardized mean difference (SMD) with 95% confidence interval (95% CI) using Comprehensive Meta-Analysis 3.0.

**Results:**

Nineteen RCTs, reporting 858 PwMS, were included. Our findings reported that VRBT is effective in improving functional balance (SMD = 0.8; 95%CI 0.47 to 1.14; *p* < 0.001); dynamic balance (SMD = − 0.3; 95%CI − 0.48 to − 0.11; *p* = 0.002); postural control with posturography (SMD = − 0.54; 95%CI − 0.99 to − 0.1; *p* = 0.017); confidence of balance (SMD = 0.43; 95%CI 0.15 to 0.71;* p* = 0.003); and in reducing fear of falling (SMD = − 1.04; 95%CI − 2 to − 0.07; *p* = 0.035); but not on gait speed (SMD = − 0.11; 95%CI: − 0.35 to 0.14; *p* = 0.4). Besides, the most adequate dose of VRBT to achieve the greatest improvement in functional balance was at least 40 sessions, five sessions per week and 40–45 min per sessions; and for dynamic balance, it would be between 8 and 19 weeks, twice a week and 20–30 min per session.

**Conclusion:**

VRBT may have a short-term beneficial role in improving balance and reducing fear of falling in PwMS.

**Supplementary Information:**

The online version contains supplementary material available at 10.1186/s12984-023-01174-z.

## Introduction

Multiple sclerosis (MS) is an immune-mediated, inflammatory and neurodegenerative chronic disease that causes demyelination and axonal degeneration in the Central Nervous System (CNS) [1]. MS has been reported as the main cause of non-traumatic disability in adults between 20 and 40 years, affecting approximately 2.8 million people in the world in 2020 [2], mainly women [3]. Patients with MS (PwMS) present motor and sensory disturbances (vestibular and visual), producing balance disorders, that are one of the most disabling consequences, affecting approximately 75% of the cases [4]. It has been reported that balance requires vestibular, visual and somatosensory inputs (both proprioceptive and exteroceptive information) [5]. Therefore, vestibular deficits (vertigo or dizziness, for example) [6], visual impairments (such as, diplopia) [7], and proprioceptive disorders due to fatigue and muscle weakness [8] may impair balance in PwMS [9]. All these issues affect postural control and different balance domains (functional, static or dynamic), reducing patients’ confidence in their balance and increasing the risk and fear of falling [10]. Balance disorders have been associated with a higher risk of falls [11]. A cross-sectional descriptive study conducted by Finlayson et al. (2006) in USA, reported that more than 50% of PwMS between 45 and 90 years old have experienced a fall in the first 6 months of the illness [12], leading to additional disabilities related to bone injuries. MS also affects gait skills by reducing gait cadence and speed, and leads to insecure gait and a greater risk of falls during the double support phase [13], with an increase of fall-related injuries and associated disability. Therefore, the fear of falling reduces the functional independence of PwMS, and their social and work relationships, restricting their quality of life [14].

In the field of neurorehabilitation, along with pharmacological and conventional therapy (CT), virtual reality-based therapy (VRBT) is being employed in the last decade to reduce the impact of disabling sequelae and to improve the quality of life of these patients [15] and others CNS disorders, such as stroke [16]. In addition, virtual reality devices are being used as a cheap diagnostic tools to assess balance disorders in these patients [17], becoming a good alternative in contrast to more expensive and sophisticated technologies. VRBT is based on the partial or total immersion of patients, through specialized software and hardware, in two- or three-dimensional virtual environments that the patients can identify as similar to the real world and with which they can interact through a manual controller (joysticks, trackpads, or trackballs) or with their bare hands [18, 19]. Depending on the level of exposure and presence in the virtual environment, there are several VRBT modalities (non-immersive VRBT [niVRBT], semi-immersive VRBT and immersive VRBT [iVRBT]). On the one hand, niVRBT is based in the use of computers or game-stations, which allow the patients to visualize and interact with the bidimensional environments projected onto a screen, using devices like keyboards, mice, and manual controllers [20, 21]. On the other hand, iVRBT provides a 360° immersion with great realism, through head-mounted display [22]. In addition, semi-immersive VR consist in the use of high speed computer that overlays virtual and tridimensional images onto real environments using three superimposed panoramic screens in front of the individual [23]. Semi-immersive VR represents a midpoint of immersion and presence between niVRBT and iVRBT being a VRBT modality recommended due to its association with fewer adverse effects, such as cybersickness [24]. Traditionally, niVRBT technologies have been more accessible and cheaper than iVR and it is being the VRBT modality most commonly used in neurorehabilitation. It has been proposed that VRBT promotes neuroplasticity, and maximizes motor learning, becoming an excellent tool for PwMS rehabilitation. VRBT may be more suitable for working on functional activities in a playful and motivating way through videogames or the recreation of virtual scenarios. This allows the patient to train numerous functional or sports activities in the same physiotherapy center or at home supervised by a physiotherapist (tele-physiotherapy or tele-rehabilitation) [25], which would increase the frequency of patient rehabilitation and could shorten recovery times [26]. Finally, VRBT is especially suitable for developing personalized functional exercises that integrate multisensory inputs aimed to restore patients’ performance on activities of daily living (ADLs) [27]. And regarding its use as a therapeutic approach in PwMS, recent studies have reported high levels of acceptance, motivation, satisfaction and adherence to the therapy [28].

In recent years, several reviews have analyzed the effect of VRBT on balance and/or risk of falls [29–34]. However, all of these reviews included a low number of studies, with the meta-analysis of the Casuso-Holgado containing the larger number studies, 11 in total [31]. Therefore, the generalization of their findings is low, and it is necessary to update these finding including new studies through a sensitive search. Functional and dynamic balance were the main domains assessed in these reviews, although a low number of studies per outcome were included. However, a relevant outcome such as confidence of balance has not been assessed in any review. Finally, none review provides data regarding the most effective dose of VRBT (number of treatment sessions or days per week) for balance treatment, or the effect observed according to the disability status of PwMS. Therefore, this meta-analysis is aimed at gathering the best available knowledge about the effectiveness of VRBT on functional and dynamic balance, postural control, confidence of balance, fear of falling and gait speed in PwMS, compared to other therapeutic approaches. The second goal of this review is aimed at determining the optimal dosing strategy for VRBT to achieve the best results in balance outcome measures (number of sessions, sessions per week and duration of each session).

## Methods

### Review protocol

A systematic review with meta-analysis was performed in accordance with the Preferred Reporting Items for Systematic Reviews and Meta-Analyses (PRISMA) 2020 statement [35]*.* It was registered in the PROSPERO database (CRD42021256768).

### Search strategy

A search was performed, independently, by two authors (ICP and FANE), in PubMed Medline, Web of Science (WOS), Scopus, CINAHL Complete and PEDro (Physiotherapy Evidence Database) and through the references of the retrieved records (previous published reviews, congress abstracts or practice guidelines, from the beginning of the database to September 30th, 2021. The search question followed the PICOS framework [36]: Population (PwMS), Intervention (VRBT), Comparison (other therapies), Outcomes (functional and dynamic balance, postural control using posturography, balance confidence, fear of falling and gait speed) and Study (randomized controlled trials [RCTs]). We designed a sensitive search strategy using the following keywords: “multiple sclerosis”, “virtual reality” and “virtual reality exposure therapy”, and entry terms, which were combined with the boolean operators “and”/“or”. No filters for publication date and language were used. A third author (EOG) with experience in search strategy provided support at this stage. Table 1 shows the search strategy.

**Table 1:** Bibliographic search strategy in each database

Databases	Search strategy
PubMed Medline	(multiple sclerosis[mh] or multiple sclerosis[tiab] or “multiple sclerosis”[tiab] or esclerosis multiple[tiab]) AND (virtual reality[mh] OR virtual reality[tiab] OR virtual reality exposure therapy[mh] OR virtual reality exposure therapy[tiab] OR exergam*[tiab] or videogam*[tiab])
Web of Science	TOPIC: (*multiple sclerosis* OR *esclerosis múltiple*) AND TOPIC: (*virtual reality* OR *exergame*)
SCOPUS	(TITLE-ABS-KEY ("multiple sclerosis" OR " esclerosis múltiple") AND TITLE-ABS-KEY ("virtual reality" OR "exergames" OR "videogames"))
PEDRO	Multiple Sclerosis AND virtual reality Multiple Sclerosis AND exergames
CINAHL	AB (multiple sclerosis OR esclerosis multiple) AND AB (virtual reality OR exergames OR videogames)

### Study selection: inclusion and exclusion criteria

Two blinded reviewers (ICP and EOG), independently, screened the titles and abstracts of all the retrieved studies for further examination. Disagreements were resolved by a third author (FANE).

The following inclusion criteria were used: (1) RCTs or pilot RCTs with at least two groups; (2) assessing the effect of VRBT on the outcomes of interest (see outcomes section) in comparison to others controls; (3) in PwMS; (4) and studies that provided quantitative data about the outcomes to perform the meta-analysis. The exclusion criteria were: (1) RCTs including patients with different neurological diseases apart from PwMS in the same group; (2) studies reporting statistical data which cannot be meta-analyzed with our software.

### Data extraction

For each study the following data were extracted: (1) overall study characteristics (authorship, publication date, country and study design); (2) number of groups; (3) sample characteristics for each group (sample size, age, gender, disability status and time since MS diagnosis); (4) characteristics of the VRBT intervention (type of VRBT, number of sessions, sessions per week and duration of each session in minutes); (5) type of therapy used as control; (6) quantitative results for each variable at the end of the intervention (mean and standard deviation [SD], or interquartile range, range and standard error to estimate the SD) [37]; and evaluation time (just at the end of the intervention or in the follow-up assessment). Data were gathered independently by two authors (ICP and DMC) using a standardized Microsoft Excel data sheet designed for this research. Disagreements were resolved by a third author (EOG).

### Variables

The variables assessed in this systematic review were three: postural balance, fear of falling and gait speed. Considering that postural balance is a complex function integrated by some dimensions, we independently assessed the functional balance, dynamic balance, confidence of balance and postural control measured with posturography.

### Analysis of risk of bias, methodological quality and evidence

Risk of bias and methodological quality of the included studies was assessed using the PEDro Scale. This scale is composed by 11 binary items (“yes” if the criterion is met or “no” when the criterion is not met) [38]. The total score is the sum of responses to items 2 to 11 (item 1 is not added to the total score since it only reports external validity), and ranges from 0 (high risk of bias) to 10 (low risk of bias) [39].

The GRADE (Grading of Recommendations Assessment, Development, and Evaluation) approach was employed to assess the level of quality evidence of findings in each meta-analysis, through the assessment of risk of bias, inconsistency, inaccuracy, indirectness and risk of publication bias [40]. With the exception of risk of bias, the checklist proposed by Meader [41] was used for assessing inconsistency (calculating the level of heterogeneity), inaccuracy (according to the number of participants per study and the number of studies per meta-analysis), indirectness (indirect evidence exists in those articles in which the results are measured indirectly, assessed as “yes” or “no”) and risk of publication bias [37]. Finally, the combination of these items allowed to establish four levels of evidence: (1) high: the findings are robust; (2) moderate: when there is the possibility that further research may change the results; (3) low; when the level of confidence in the pooled effect is very modest; or (4) very low: any estimate of the effect is highly uncertain. Risk of bias and quality evidence assessment were performed by two authors (ICP and RLV), with the support a third author (FANE).

### Statistical analysis

Statistical analysis was performed by two authors by using *Comprehensive Meta-Analysis version 3.0* (Biostat, Englewood, NJ, USA) [42]. A meta-analysis was done only when more than one study provided data about an outcome. The pooled effect was calculated using the Cohen’s standardized mean difference (SMD) [43] with 95% confidence interval (95% CI) in a random-effects model [44]. SMD provides four effect strength levels: no effect (SMD 0), small (SMD 0.2), medium (SMD 0.5) and large (SMD ≥ 0.8) [45]. In addition, for outcomes assessed using the same measure we calculated the Mean Difference (MD) between groups in order to compare our results with the Minimal Clinically Important Difference (MCID) value for such test. The pooled effect was displayed through forest plots [46]. The risk of publication bias was assessed according to the symmetry (low risk) or asymmetry (high risk) of the funnel plot [47] using the Egger’s test (where if *P* < 0.1 there exists a risk of publication bias) [48]. In addition, the Trim-and-fill method was used to estimate the adjusted SMD, taking into account any possible risk of publication bias [49]. According to Rothman’s recommendations for the effect size variation limit in the assessment of confusion bias, when the adjusted SMD varied more than 10% with respect to the original and raw pooled effect, the quality level of evidence was downgraded one level, although the funnel plot was slightly asymmetrical [50]. Finally, the level of heterogeneity was assessed with the *P* for Q-test and the degree of inconsistency (*I*^*2*^) from Higgins [51]. Heterogeneity may exist when *P* < 0.1 and it can be categorized as low (*I*^*2*^ < 25%), moderate (*I*^*2*^ 25–50%) or large (*I*^*2*^ > 50%) [37, 51].

### Additional statistical analysis

In order to assess the contribution of each study to the global effect in each meta-analysis, a sensitivity analysis was performed using the leave-one-out method [37]. In addition, different subgroup analyses were performed. The first subgroup analysis was done for the comparisons carried out in the RCTs: VRBT vs usual care (UC); VRBT vs conventional therapy (CT, physical therapy); VRBT + CT vs CT; and VRBT + Robotic assisted gait training (RAGT) vs RAGT). The second subgroup analysis was carried out according to patients’ disability status assessed with the Kurtzke’s Expanded Disability Status Scale (EDSS) [52]. This scale provides information about the disability status of PwMS with the aim of being used by health care clinicians in the diagnosis and management of MS. EDDS classifies disability status in 20 scores from 0 (normal neurological exam and no disability) to 10 (death due to MS). According to the 20 possible scores provided by Kurtzke, JF (1983) [52], we carried out a more simplified and functional reorganization of this scale, identifying the following subgroups: Only minimal disability = EDDS 0.5; minimal disability = EDDS 1–2.5; moderate disability = EDDS 3–3.5; severe disability = EDDS 4–4.5; and disability affects ADL = EDDS 5–5.5). Finally, the third subgroup analysis was performed to estimate the most appropriate dose of VRBT according to: number of sessions of VRBT (8–19, 20–39, ≥ 40 sessions); number of sessions per week of VRBT (1, 2, 3, 4 or 5 sessions per week); and duration of each VRBT session in minutes (20–30, 40–45 or 60 min).

## Results

### Study selection

**Fig. 1:**
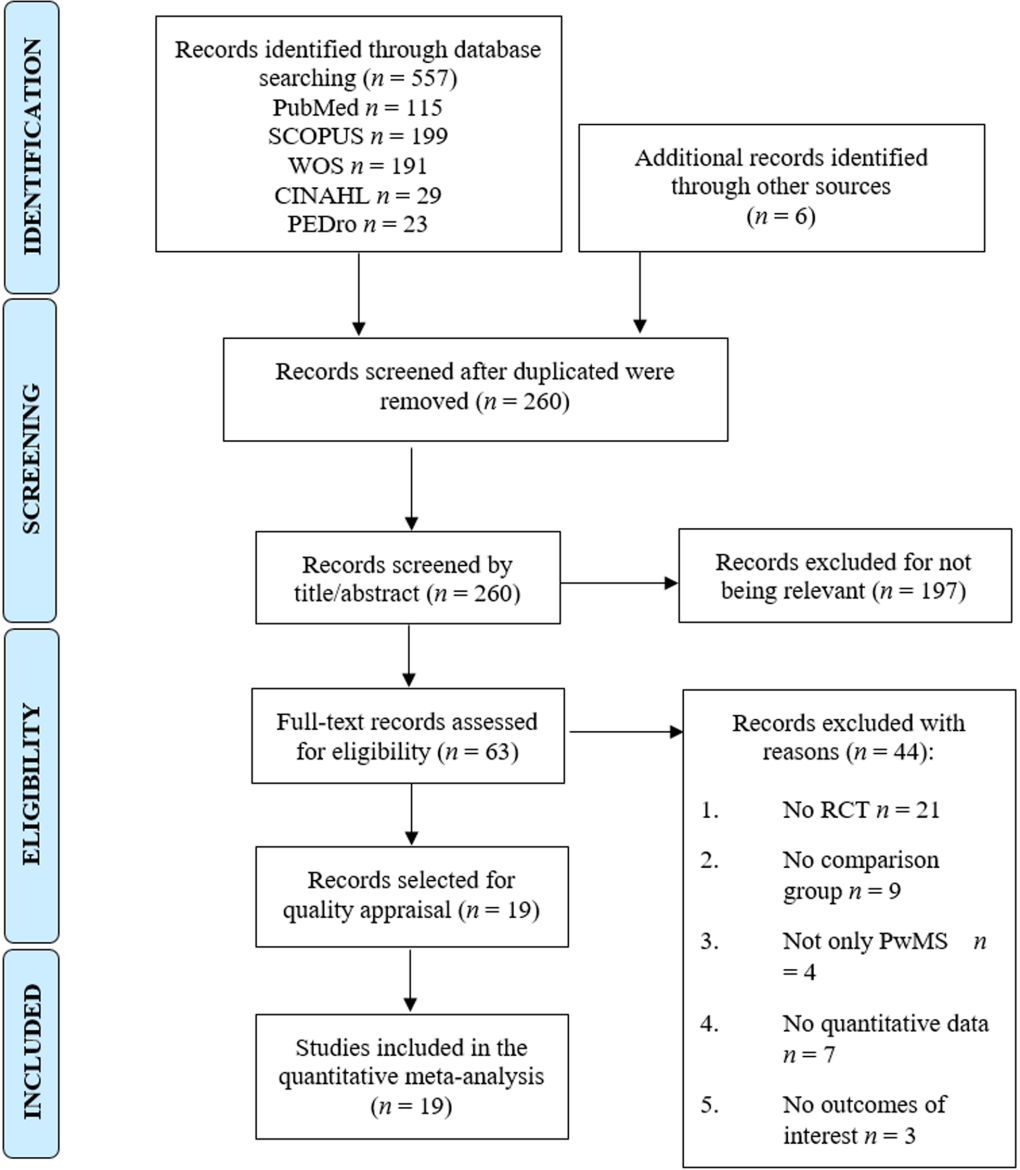
PRISMA flow chart of the study selection process

Five hundred and sixty-three references were identified (557 studies from databases and 6 retrieved after a manual search in the references of full-text screened studies and other sources). After removing duplicate records (*n* = 303), 260 studies were screened by title/abstract. One hundred and ninety-seven studies were excluded by title/abstract whereas 44 did not meet the inclusion criteria. Finally, 19 RCTs [53–71] were included in this review. Figure 1 shows the PRISMA flow chart of the study selection process.

### Characteristics of the studies included in the review

The included RCTs were carried out in the last 10 years (2012 [70], 2013 [53, 57, 71], 2014 [66], 2015 [63, 64], 2016 [56, 62], 2017 [54, 58], 2018 [65], 2019 [59], 2020 [55, 60, 61, 67, 69], and 2021[68]) in Italy [53, 54, 56, 57, 67, 69], Spain [66, 71], Turkey [55, 60, 61], United Kingdom [58, 63], Iran [64, 68], Sweden [70], Hungary [59], Israel [62], and Jordan [65]. These studies provided data from 858 PwMS (mean age of 43.4 ± 6.7 years old, moderate status of disability of 3.6 ± 1.2 in EDSS and 10.1 ± 3.3 years since diagnosis). According to their sex, 606 PwMS were women (approximately 71%) and 252 were males. A total of 441 PwMS (43.7 ± 7.6 years old) were included in the experimental group and received VRBT using niVRBT [53, 54, 56–62, 64–66, 68–71], iVRBT [55] and semi-iVRBT systems [67]; on the other hand, the control group included 417 PwMS (43.1 ± 5.7 years old). We identified the following therapy comparisons in the included studies: VRBT vs UC in 8 studies [55, 57, 59–61, 63, 64, 70]; VRBT vs CT in 10 studies [53, 55, 59–63, 65, 68, 71]; VRBT + CT vs CT in 3 studies [58, 66, 67]; and VRBT + RAGT vs RAGT in other 3 studies [54, 56, 69]. The number of VRBT sessions received by the participants in the experimental group was heterogeneous, ranging from 8 to 60 sessions; and the number of sessions per week varied between 1 and 5 sessions. The meta-analysis showed an effect of VRBT in the short-term. Table 2 summarizes the main characteristics of the included RCTs.

**Table 2:** Main characteristics in studies included in the meta-analysis

				Virtual reality group									Control group					Comparison
				Sample characteristics				VRBT characteristics					Sample characteristics					
Authorship and publication date	Design	Country	N	N_i_	Age	EDSS	Diag. Time	Type	Ses	Week	Ses/week	Min	N_c_	Age	EDSS	Diag. Time	Type control	
Brichetto et al. 2013	Pilot RCT	Italy	36	18	40.7	3.9	11.2	niVRBT	12	4	3	60	18	43.2	4.3	12.3	CT	VRBT vs CT
Calabrò et al. 2017	Single-blind RCT	Italy	40	20	44	4.4	11	niVRBT	40	8	5	40	20	41	4.75	10	RAGT	VRBT + RAGT vs RAGT
Eftekharsadat et al. 2015	Single-blind RCT	Iran	30	15	33.4	–	5.8	niVRBT	24	12	2	20	15	37	–	8.3	UC	VRBT vs UC
Kalron et al. 2016	Pilot RCT	Israel	30	15	47.3	4.5	11.6	niVRBT	18	6	2	30	15	43.9	3.9	10.4	CT	VRBT vs CT
Khalil et al. 2019	Single-blind RCT	Jordan	32	16	39.8	2.9	8.4	niVRBT	12	6	2	–	16	34.8	3.1	10.4	CT	VRBT vs CT
Lozano-Quilis et al. 2014	Single-blind RCT	Spain	11	6	48.3	–	14	niVRBT	10	10	1	60	5	40.6	–	4.7	CT	VRBT + CT vs CT
Maggio et al. 2020	Single-blind RCT	Italy	60	30	51.9	–	–	Semi-iVRBT	24	8	3	60	30	48.2	–	–	CT	VRBT + CT vs CT
Molhemi et al. 2021	Single-blind RCT	Iran	39	19	36.8	4.8	7.7	niVRBT	18	6	3	30	20	41.6	4.7	11.2	CT	VRBT vs CT
Munari et al. 2020	Pilot RCT	Italy	15	8	57	5.4	17.7	niVRBT	12	6	2	40	7	51.7	5	13.9	RAGT	VRBT + RAGT vs RAGT
Nilsagard et al. 2012	Multi-centre RCT	Sweden	80	41	50	-	12.5	niVRBT	12	6	2	30	39	49.4	–	12.2	UC	VRBT vs UC
Ortiz-Gutiérrez et al. 2013	Single-blind RCT	Spain	47	24	36	3.95	9.7	niVRBT	40	10	4	20	23	42.7	3.8	10.8	CT	VRBT vs CT
Ozkul et al. 2020	Single-blind RCT	Turkey	39	13	29	1	4	iVRBT	16	8	2	20	13	34	1	4	CT	VRBT vs CT
													13	34	2	4	UC	VRBT vs UC
Peruzzi et al. 2016	Single-blind RCT	Italy	25	14	43.6	4.1	11.8	niVRBT	18	6	3	30	11	42	3.5	12.4	RAGT	VRBT + RAGT vs RAGT
Prosperini et al. 2013	Cross-over RCT	Italy	36	18	35.3	3	12.2	niVRBT	60	12	5	30	18	37.1	3.5	9.3	UC	VRBT vs UC
				18	37.1	3.5	9.3	niVRBT	60	12	5	30	18	35.3	3	12.2	UC	VRBT vs UC
Robinson et al. 2015	Prospective RCT	UK	51	20	52.6	–	–	niVRBT	8	4	2	40–60	16	53.9	–	–	CT	VRBT vs CT
													15	51.9	–	–	UC	VRBT vs UC
Thomas et al. 2017	Single-blind RCT	UK	30	15	50.9	–	–	niVRBT	–	–	–	–	15	47.6	–	–	CT	VRBT + CT vs CT
Tóllar et al. 2019	Single-blind RCT	Hungary	50	14	48.2	5	12.1	niVRBT	25	5	5	60	14	46.9	5	13.6	CT	VRBT vs CT
													12	47	5	13.1	UC	VRBT vs UC
Tuba-Ozdogar et al. 2020	Single-blind RCT	Turkey	57	20	39.2	2.7	7.5	niVRBT	8	8	1	45	17	43.6	2.1	6.4	CT	VRBT vs CT
													20	37.9	2.3	5.9	UC	VRBT vs UC
Yazgan et al. 2020	Single-blind RCT	Turkey	42	15	47.4	4.16	12.06	niVRBT	16	8	2	60	12	43.1	3.8	14.9	CT	VRBT vs CT
													15	40.6	4	11	UC	VRBT vs UC

*N* total sample size in each study, *Ni* sample size in intervention group, *EDSS* Kurtzke’s Expanded Disability Status Scale, *Diag. Time* time since diagnosis in years, *Ses* number of sessions, *Min* minutes, *Nc* sample size in control group, *RCT* randomized controlled trial, *niVRBT* non-immersive virtual reality-based therapy, *Semi-iVRBT* semi-immersive virtual reality-based therapy, *iVRBT* immersive virtual reality-based therapy, *CT* conventional therapy, *UC* usual care or simple observation, *RAGT* Robotic Assisted Gait Training, *UK* United Kingdom

### Risk of bias and methodological quality assessment

The mean PEDro score was 6.2 ± 1, showing a moderate quality of the included studies. Six studies [53, 55, 60, 61, 63, 66] showed low methodological quality, 11 studies [57–59, 62, 64, 65, 67, 69–71] moderate, and 2 studies [54, 68] high quality scores. The impossibility of blinding participants and therapists favors the presence of performance and detection biases, respectively, in all studies. In addition, selection bias can appear in studies in which the item of “concealed allocation” is not met. Table 3 shows the PEDro score for each RCT.

**Table 3:** PEDro scores for methodological assessment of the studies included in the review

Study	Items											
	1	2	3	4	5	6	7	8	9	10	11	Total
Brichetto et al. 2013	Yes	Yes	No	Yes	No	No	Yes	No	No	Yes	Yes	5/10
Calabrò et al. 2017	Yes	Yes	Yes	Yes	No	No	Yes	Yes	Yes	Yes	Yes	8/10
Eftekharsadat et al. 2015	Yes	Yes	No	Yes	No	No	Yes	Yes	No	Yes	Yes	6/10
Kalron et al. 2016	No	Yes	Yes	Yes	No	No	Yes	Yes	No	Yes	Yes	7/10
Khalil et al. 2018	Yes	Yes	Yes	Yes	No	No	Yes	No	No	Yes	Yes	6/10
Lozano-Quilis et al. 2014	Yes	Yes	No	Yes	No	No	No	Yes	No	Yes	Yes	5/10
Maggio et al. 2020	Yes	Yes	Yes	Yes	No	No	Yes	Yes	No	Yes	Yes	7/10
Molhemi et al. 2021	Yes	Yes	Yes	Yes	No	No	Yes	Yes	Yes	Yes	Yes	8/10
Munari et al. 2020	Yes	Yes	Yes	Yes	No	No	Yes	Yes	No	Yes	Yes	7/10
Nilsagard et al. 2012	Yes	Yes	Yes	Yes	No	No	Yes	Yes	No	Yes	Yes	7/10
Ortiz-Gutiérrez et al. 2013	Yes	Yes	No	Yes	No	No	Yes	Yes	No	Yes	Yes	6/10
Ozkul et al. 2020	Yes	Yes	No	Yes	No	No	No	Yes	No	Yes	Yes	5/10
Peruzzi et al. 2016	Yes	Yes	No	Yes	No	No	Yes	No	No	Yes	Yes	5/10
Prosperini et al. 2013	Yes	Yes	Yes	Yes	No	No	No	Yes	No	Yes	Yes	6/10
Robinson et al. 2015	Yes	Yes	No	Yes	No	No	No	No	Yes	Yes	Yes	5/10
Thomas et al. 2017	Yes	Yes	Yes	Yes	No	No	No	Yes	Yes	Yes	Yes	7/10
Tóllar et al. 2019	Yes	Yes	Yes	Yes	No	No	Yes	Yes	No	Yes	Yes	7/10
Tuba-Ozdogar et al. 2020	No	Yes	No	Yes	No	No	No	Yes	No	Yes	Yes	5/10
Yazgan et al. 2020	Yes	Yes	No	Yes	No	No	No	Yes	No	Yes	Yes	5/10

1: Eligibility criteria, 2: Random allocation, 3: Concealed allocation, 4: Baseline comparability, 5: Blind subjects, 6: Blind therapists, 7: Blind assessors. 8: Adequate follow-up, 9: Intention-to-treat analysis, 10: Between-group comparisons, 11: Point estimates and variability. Note: Eligibility criteria item does not contribute to total score

### Outcomes synthesis

We identified different balance domains in the included RCTs and different meta-analysis were performed according to each dimension. Functional balance was assessed by using quantitative data from the Berg Balance Scale (BBS) [72] and dynamic balance from the Timed Up & Go-Test (TUGT) [73] and the Four Square Step Test (4SST) [74]. Thirteen RCTs provided quantitative data from the BBS assessment [53–56, 59, 61, 62, 64–66, 68, 69, 71], 11 RCTs from the TUGT [54–56, 58, 61, 64–66, 68, 70, 75] and 2 RCTs from the 4SST [57, 62]. Secondly, Postural control was assessed by mean of posturography assessment, using Sway Area [53] and Center of Pressure (CoP) excursion [62], both for eyes open (EO) and closed (EC). Three RCTs provided quantitative data about Sway Area [53, 62, 69] and 5 RCTs about CoP excursion analyses [57, 60, 62, 63, 69]. Thirdly, confidence of balance was assessed through quantitative data from the Activities-Specific Balance Confidence (ABC) scale [76] obtained from 3 RCTs [60, 68, 70], whereas fear of falling was assessed through the Falls Efficacy Scale (FES-1) [77] reported in 3 RCTs [62, 65, 68]. Finally, gait speed was assessed using data from the 10 Meters’ Walk test (10MWT) [78] and the Timed 25-Foot Walk Test (25FWT) [79]. Five RCTs provided quantitative data from the 10MWT assessment [56, 65, 66, 68, 69], and 2 RCTs from the 25FWT [57, 60].

### Quantitative synthesis

All studies were included in the quantitative synthesis. Table 4 summarizes the main findings in the meta-analysis of each variable.

**Table 4:** Main findings in meta-analyses

	Summary of findings									Grade quality evidence					
	Effect size heter						Publication bias								
	K	N	N_s_	SMD	95% CI	I^2^ (p-value)	funnel plot (Egger p-value)	trim and fill		Risk of bias	Incons	Indirect	Imprec	Publication bias	Quality evidence
								Adj SMD	% var (%)						
Functional balance	16	451	28.1	0.8	0.47 to 1.14	0% (p = 0.47)	Slightly Asym (p = 0.89)	0.94	16	Medium	No	No	Yes	Probable	Moderate
Dynamic balance	16	494	30.8	− 0.3	− 0.48 to − 0.11	3.7% (p = 0.48)	Asym (p = 0.24)	− 0.18	42	Medium	No	No	Yes	Yes	Low
Sway area EC	3	81	27	− 0.54	− 0.99 to − 0.1	0% (p = 0.55)	Slightly Asym (p = 0.27)	− 0.54	0	Medium	No	No	Yes	No	Very-low
Centre of pressure escursion EO	8	189	23.6	− 0.25	− 0.5 to − 0.01	0% (p = 0.99)	Asym (p = 0.48)	− 0.26	8	Medium	No	No	Yes	Probable	Low
Confidence of balance	4	176	44	0.43	0.15 to 0.71	0% (p = 0.93)	Slightly Asym (p = 0.51)	0.43	0	Medium	No	No	Yes	No	Low
Fear of falling	3	101	33.6	− 1.04	− 2 to − 0.07	14.2% (p = 0.31)	Slightly Asym (p = 0.1)	− 1.04	0	Medium	No	No	Yes	No	Low
Gait speed	9	251	27.8	− 0.11	− 0.35 to 0.14	0% (p = 0.53)	Asym (p = 0.12)	− 0.06	46	Medium	No	No	Yes	Yes	Low

*Heter *heterogeneity, *K* number of comparisons, *N* number of participants in each meta-analysis, *N*_***s***_ number of participants per study, *SMD* standardized mean difference, *95% CI* 95% confidence interval, *I*^*2*^ degree of inconsistency, *Adj* adjusted, *% var* percentage of variation, *Indirect* indirectness, *Imprec* imprecision, *Asym* asymmetric, *EC* closed eyes, *EO* eyes open

### Effect of VRBT on functional balance

**Fig. 2:**
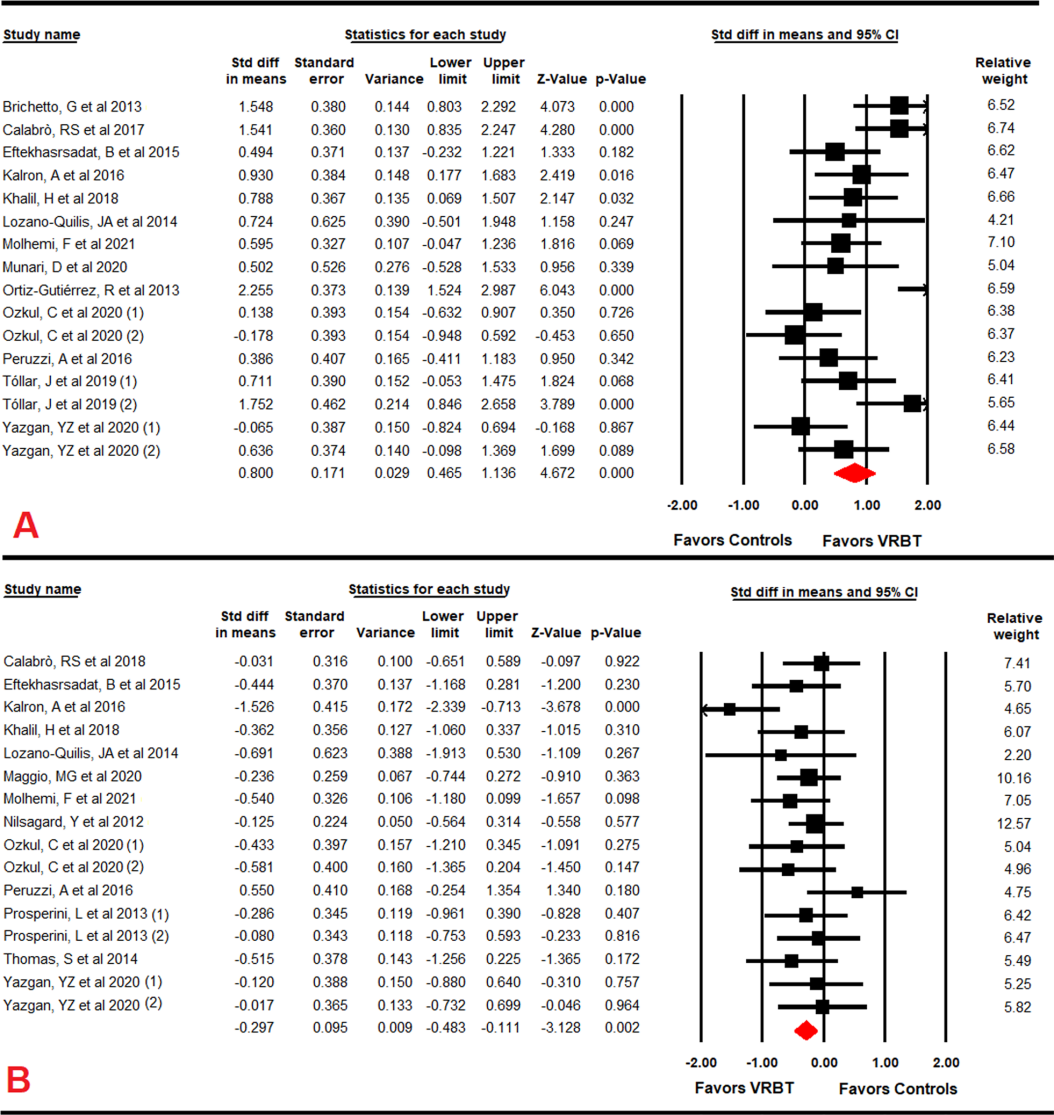
Forest plot of the effect of virtual reality-based therapy on functional (**A**) and Dynamic Balance (**B**)

Thirteen RCTs [53–56, 59, 61, 62, 64–66, 68, 69, 71] provided data to assess the efficacy of VRBT to improve functional balance. Our findings reported a moderate-quality evidence with a large effect size (SMD = 0.8; 95% CI 0.47 to 1.14; *p* < 0.001) in favor of VRBT (Table 4; Fig. 2A). In addition, an increase of 3.36 points (95% CI 2.26 to 4.48; *p* < 0.001) on BBS is observed in favor of VRBT compared to other controls. A possible risk of publication bias has been identified (Egger *p* = 0.9 and Trim-and-fill variation of 16%) (Additional file 2: Fig. S1) without heterogeneity (I^2^ = 0%; *p* = 0.47). Sensitivity analysis did not show variations.

For specific subgroups comparisons, the analyses of the BBS show: an increase of 3.92 points (n = 4; 112 PwMS; 95% CI 1.2 to 6.7; *p* = 0.005) in favor of VRBT when compared with UC [55, 59, 61, 64]; an increase of 3.4 points (n = 8; 265 PwMS; 95% CI 1.77 to 5; *p* = 0.001) in favor of VRBT when compared with CT [53, 55, 59, 61, 62, 65, 68, 71]; and an increase of 3.03 points (n = 3; 80 PwMS; 95% CI 0.7 to 5.38; *p* = 0.011) in favor of VRBT + RAGT vs RAGT [54, 56, 69]. The subgroup analysis also revealed that the major improvement on functional balance (n = 2; 83 PwMS; SMD = 1.91; 95% CI 1.19 to 2.63; *p* < 0.001) was observed in PwMS with moderate disability (Additional file 1: Table S2) [53, 71]. Additionally, the maximal functional balance improvement in PwMS following VRBT protocols requires: at least 40 sessions (n = 2; 87 PwMS; SMD = 1.9; 95% CI 1.2 to 2.59; *p* < 0.001) [54, 71]; being five times per week the most effective schedule (n = 2; 94 PwMS; SMD = 1.31; 95% CI 0.76 to 1.86; *p* < 0.001) [54, 59]; and 40–45 min per session the optimal duration (n = 2; 55 PwMS; SMD = 1.1; 95% CI 0.02 to 2.15; *p* = 0.045) [54, 69] (Additional file 1: Tables S3, S4 and S5).

### Effect of VRBT on dynamic balance

Data from thirteen RCTs [54–58, 61, 62, 64–66, 68, 70, 75] were used to analyze the efficacy of VRBT to improve dynamic balance. Our results showed a low-quality evidence with a small effect size of VRBT (SMD = − 0.3; 95% CI − 0.48 to − 0.11; *p* = 0.002) on dynamic balance in favor of VRBT (Table 4; Fig. 2B). A high risk of publication bias was observed (Egger *p* = 0.24 and Trim-and-fill variation of 40%) (Additional file 2: Fig. S2) but no heterogeneity (I^2^ = 3.7%; *p* = 0.48). Sensitivity analysis showed a variation of 19% in the effect size with respect to the original SMD when the study of Kalron [62] was removed, although the effect direction of the outcome did not change (SMD = − 0.23; 95% CI − 0.41 to − 0.6; *p* = 0.008).

Compared to CT, the analysis showed a medium effect of VRBT (n = 5; 179 PwMS; SMD = − 0.56; 95% CI − 0.89 to − 0.24; *p* = 0.001) in favor of VRBT [55, 61, 62, 65, 68]. The subgroup analysis also revealed a larger effect of VRBT on the dynamic balance of PwMS presenting minimal signs of disability (n = 1; 39 PwMS; SMD = − 0.51; 95% CI − 1.29 to − 0.28; *p* = 0.049) [55] (Additional file 1: Table S2). Besides, the parameters to get the major improvement in dynamic balance in PwMS were: between 8 and 19 sessions (n = 8; 326 PwMS; SMD = − 0.35; 95% CI − 0.61 to − 0.07; *p* = 0.012) [55, 56, 61, 62, 65, 66, 68, 70]; 2 sessions per week (n = 6; 281 PwMS; SMD = − 0.4; 95% CI − 0.68 to − 0.11; *p* = 0.007) [55, 61, 62, 64, 65, 70]; and a duration of 20–30 min per session (n = 7; 328 PwMS; SMD = − 0.36; 95% CI − 0.62 to − 0.1; *p* = 0.01) [55–57, 62, 64, 68, 70] (Additional file 1: Tables S3, S4 and S5).

### Effect of VRBT on postural control

**Fig. 3:**
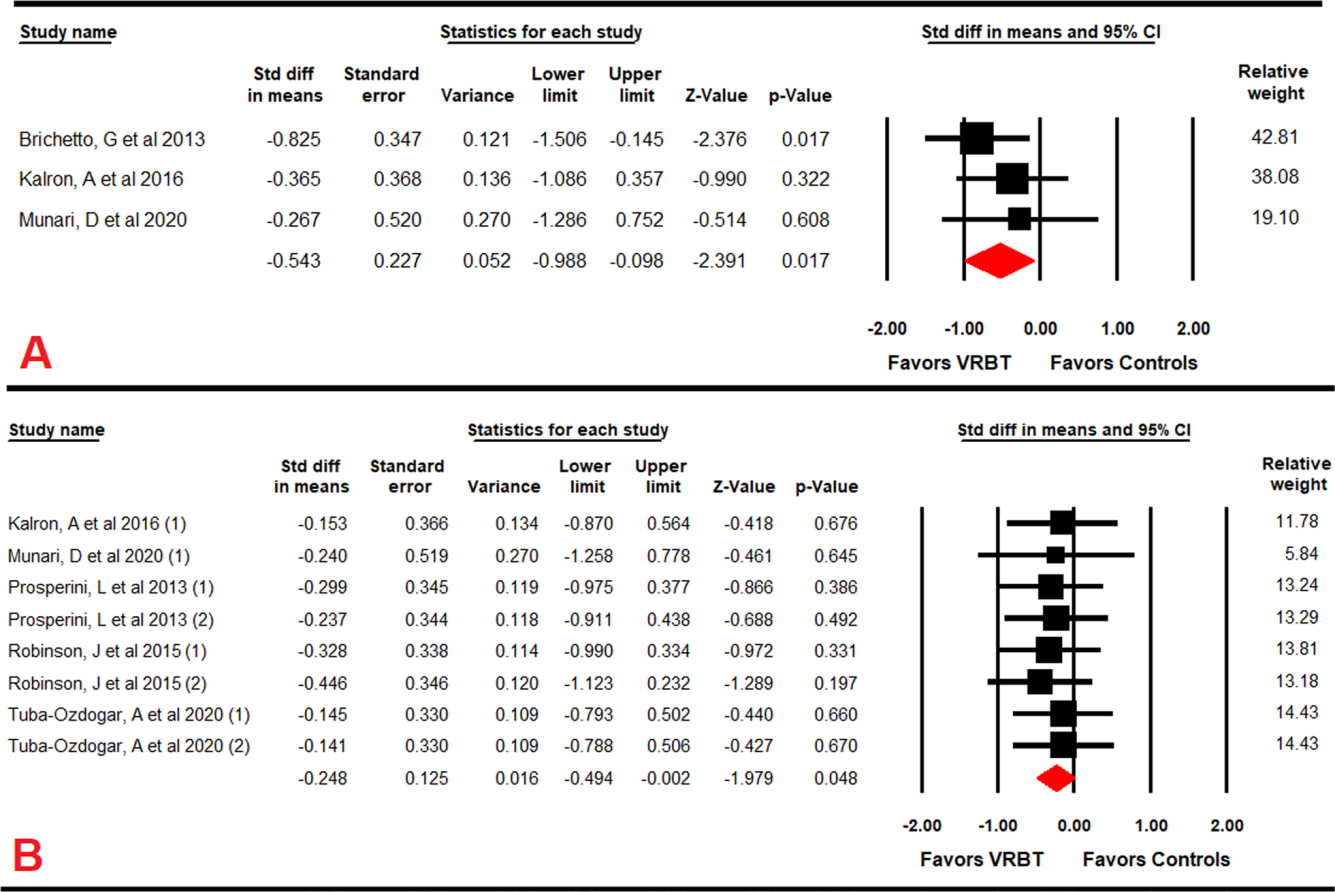
Forest plot of the effect of virtual reality-based therapy on sway area with eyes closed (**A**) and CoP with eyes open (**B**)

Three studies [53, 62, 69] provided data to assess the effect of VRBT on postural control assessed with posturography (Sway Area for eyes open (EO) and eyes closed (EC) conditions). Very low-quality evidence with a medium size effect in favor of VRBT (SMD = − 0.54; 95% CI − 0.99 to − 0.1; *p* = 0.017) was observed on Sway Area for the EC condition (Table 4; Fig. 3A), without risk of publication bias or heterogeneity (I^2^ = 0%; *p* = 0.55) (Additional file 2: Fig. S3). A medium size effect was also found in favor of the VRBT vs CT to improve Sway Area in the EC condition (SMD = − 0.61; 95% CI − 1.1 to − 0.11; *p* = 0.004) [53, 62].

Five studies [57, 60, 62, 63, 69] reported data to analyze the effect of VRBT on CoP excursion both for EO and EC conditions in comparison to other interventions. A low-quality evidence for a small effect size in favor of VRBT (SMD = − 0.25; 95% CI − 0.5 to − 0.002; *p* = 0.048) on CoP excursion with OE has been observed (Table 4; Fig. 3B), with no risk of publication bias or heterogeneity (I^2^ = 0%; *p* = 0.99) (Additional file 2: Fig. S4). Subgroup analysis according specific comparison, showed that low effect of VRBT in comparison UC (SMD = 0.27; 95% CI − 0.53 to − 0.001; p = 0.049).

### Effect of VRBT on confidence of balance

**Fig. 4:**
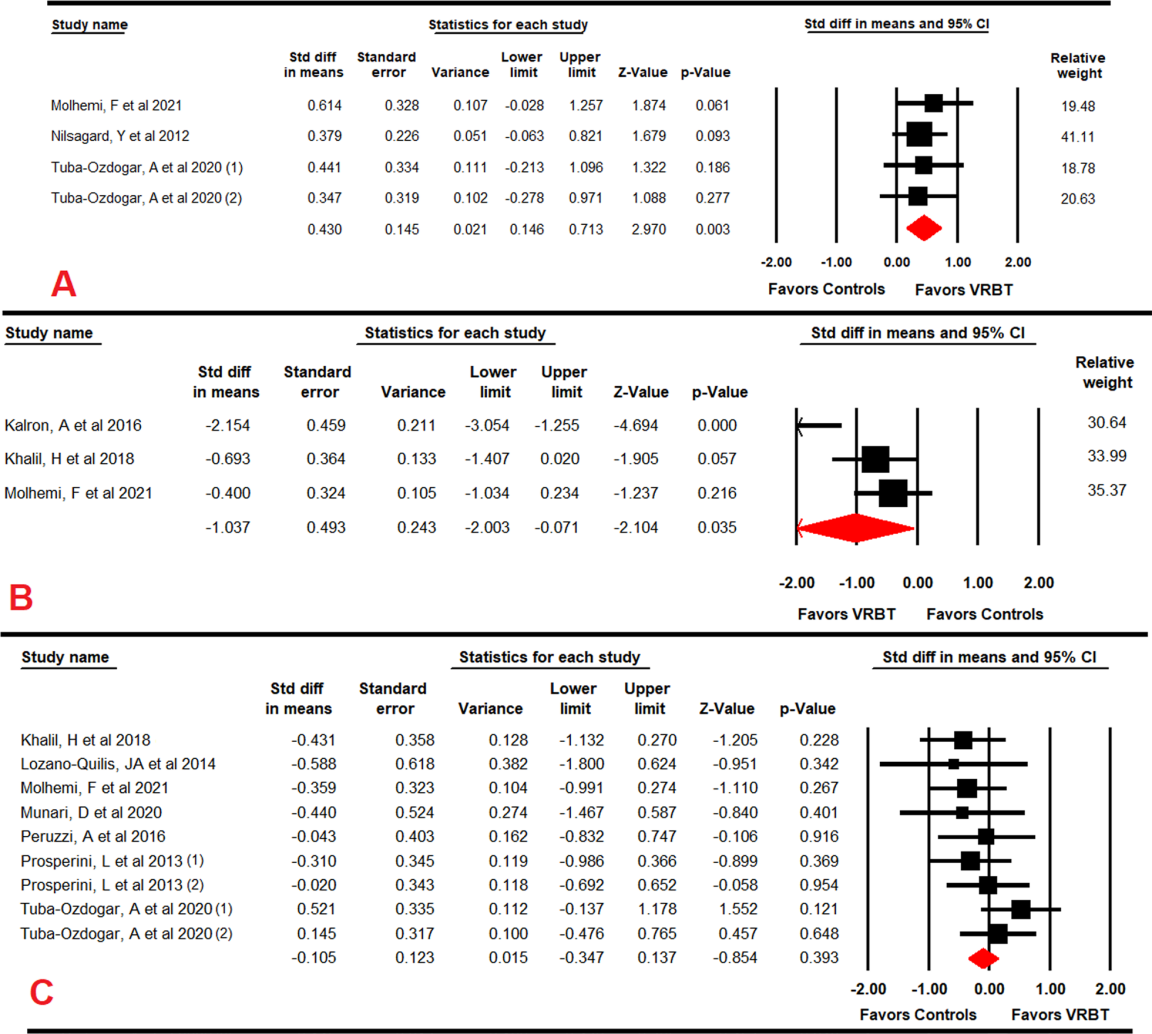
Forest plot of the effect of virtual reality-based therapy on confidence of balance (**A**), on fear of falling (**B**) and on gait speed (**C**)

Three RCTs [60, 68, 70] provided data to assess the efficacy of VRBT to increase confidence of balance. Our findings reported a low-quality evidence with a medium effect size (SMD = 0.43; 95% CI 0.15 to 0.71; *p* = 0.003) in favor of VRBT (Table 4; Fig. 4A). Confidence of balance increased by 6.81 points (95% CI 2.24 to 11.4; *p* = 0.001) on the ABC scale in favor VRBT, with no risk of publication bias (Additional file 2: Fig. S5) and without heterogeneity (I^2^ = 0%; *p* = 0.93). Sensitivity analysis did not show variations.

Subgroup analysis revealed an increase of 10.03 points (n = 2; 120 PwMS; 95% CI 1.62 to 18.44; *p* = 0.001) on the ABC scale in favor of VRBT compared to UC [60, 70], and of 5.46 (n = 2; 76 PwMS; 95% CI 0.01 to 10.92; *p* < 0.001) compared to CT [60, 68].

### Effect of VRBT on fear of falling

Three RCTs [62, 65, 68] provided data to assess the efficacy of VRBT vs CT in reducing the fear of falling, reporting a low quality evidence with a large effect in favor of VRBT (SMD = − 1.04; CI 95% − 2 to − 0.07; *p* = 0.035) (Table 4; Fig. 4B). Fear of falling was reduced by 2.86 points (95% CI − 4.1 to − 1.69; *p* = 0.002) in FES-1 in favor of VRBT. No risk of publication bias was found (Additional file 2: Fig. S6) and heterogeneity was low (I^2^ = 14.2%; *p* = 0.31). Sensitivity analysis showed a variation of 51% in the effect size after removing the study of Kalron [62], although the effect direction did not change (SMD = − 0.53; 95% CI − 1 to − 0.06; *p* = 0.03).

### Effect of VRBT on gait speed

Seven RCTs [56, 57, 60, 65, 66, 68, 69] provided data to assess the efficacy of VRBT on gait speed. Not statistically significant differences were found between VRBT and controls (SMD = − 0.11; 95% CI − 0.35 to 0.14; *p* = 0.4) (Table 4; Fig. 4C). A high risk of publication bias (Egger *p* = 0.12 and Trim-and-fill change of 46%) (Additional file 2: Fig. S7) without heterogeneity (I^2^ = 0%; *p* = 0.53) was observed. Sensitivity analysis did not report substantial variations. Subgroup analyses, according specific comparisons, did not show differences between VRBT vs UC (SMD = − 0.05; 95% CI − 0.43 to 0.33; *p* = 0.8), VRBT vs CT (SMD = − 0.08; 95% CI − 0.47 to 0.3; *p* = 0.67), and VRBT + CT vs CT (SMD = − 0.59; 95% CI − 1.8 to 0.63; *p* = 0.34).

## Discussion

The aim of this systematic review with meta-analysis was to collect all previous RCTs assessing the effectiveness of VRBT to improve balance in its different dimensions and to reduce fear of falling in PwMS. A second aim was to define the optimal dose of the VRBT protocol to improve functional and dynamic balance in PwMS. The present findings suggest that VRBT: (1) improves functional and dynamic balance; (2) increases confidence of balance and postural control assessed with posturography; (3) reduces fear of falling; (4) but, does not improve gait speed in PwMS. Compared to previous reviews [29–34], the current study provides the most comprehensive meta-analysis to date aimed at assessing the effect of VRBT on balance and its different dimensions. It includes the larger number of studies to date (19 RCTs), and the largest sample of participants (858 PwMS), which increases the robustness and generalization of its findings. In addition, the current meta-analysis includes an exhaustive analysis of subgroups, comparing the efficacy of VRBT with regard to other therapies, and according to the level of disability in PwMS.

The present results have shown that VRBT is effective improving functional balance (with regard to ADLS) in PwMS. A large effect is also observed for VRBT, with an increase of 3.36 points on the BBS score when compared to other interventions or UC. Regarding functional balance results (assessed with BBS) from previous reviews, we must mention that not the all reviews found significant improvements in VRBT compared to other therapies. While Casuso-Holgado and Santos-Nascimento did not show statistically significant differences between VRBT and CT [31, 32], our findings are in line with Parra-Moreno and Calafiore, who reported an improvement on BBS in comparison to CT [30, 34]. Our results are clinically relevant regarding the effect on the BBS scale, surpassing the MCID reported by Gervasoni [80] who proposed an improvement higher than 3 points as MCID for the BSS in a sample of PwMS. In addition, subgroups analyses revealed that VRBT is better than UC and CT, and surpassed the MCID in contrast to UC, CT or RAGT. Another interesting result from the current study is that RAGT + VRBT is more effective for improving BBS score than RAGT alone. It points to the importance of using robotic systems complemented with virtual reality devices for gait training. This meta-analysis has also shown that VRBT is more efficient to improve functional balance, compared other therapies, in patients with moderate, severe and restricted ADLS. The larger effect was observed in patients with moderate disability (fully ambulatory patients with a score of 3–3.5 points in EDSS). Our findings showed that the most adequate VRBT dose to achieve the best improvement in functional balance would be at least 40 sessions, five sessions per week and 40–45 min per sessions. Although our meta-analysis has not assessed if functional activities training improves functional balance, it is advisable that VRBT includes functional exercises similar to ADLs, both in standing or sitting position. It could improve PwMS ability to maintain their balance during ADLs performance.

We must note that in contrast to the reviews by Casuso-Holgado and Santos-Nascimento [31, 32], our review shows that VRBT may be effective to increase dynamic balance in PwMS, with a small effect size. These findings indicate that, in contrast to CT, VRBT produces a low-medium size effect on dynamic balance, and points out that VRBT is superior to CT for improving dynamic balance in PwMS. Moreover, as dynamic balance requires greater mobility skills to perform ADLS in a standing position, VRBT produces a larger effect in PwMS with minimal symptoms of disability. In addition, our results show that the best VRBT protocol for improving dynamic balance requires between 8 and 19 sessions, and must be carried out 2 times per week, with a duration of 20–30 min per session. However, it has not been possible to determine if VRBT surpasses the MCID threshold for dynamic balance (TUGT) due to the variability of tests employed in the RCTs that assessed this variable (TUGT and 4SST).

Regarding the effect on postural control assessed with posturography, the meta-analysis shows a medium effect on Sway Area in EC condition, and a small effect on CoP excursion in EO in favor of VRBT compared to CT. This points out that VRBT is helpful to reorganize the sensory inputs related with balance (vestibular, visual and somatosensory). Thus, VRBT leads to an increase of postural control when different sensory inputs are lost. In addition, our findings showed that VRBT does not improve gait speed, in agree to Casuso-Holgado [31].

As shown in the review of Akkan, [33] the improvement in the perception of one's own balance increases the confidence of balance of PwMS and reduces their fear of falling. Previous studies have reported a high risk of falling (more than 53% of PwMS), and identified numerous risk factors, being the most important impaired balance, motor disability, cognitive sequelae and the type of MS diagnosed [11, 14, 81, 82]. Therefore, the improvement in functional and dynamic balance could be the reason for increased balance confidence in PwMS and the lower fear of falling during ADLs. A recent review highlights that physical exercise is an excellent and active therapeutic option to reduce the risk of falls in PwMS [83]. VRBT is an active therapy that permits to simulate different environments where PwMS can perform different physical exercises aimed to reduce the risk of falling [20].

Multisensory information is crucial to produce an effective antigravity muscular response in order to avoid destabilization and to maintain balance [84]. Therefore, it is recommendable to develop therapies that include multisensory stimulation and active work aimed at improving balance. Accordingly, VRBT combines multisensory and entertaining stimuli that help to maintain a continuous state of attention and motivation during the activity [19]. Multisensory activation may involve the mirror neurons system and promote neuroplasticity processes in unaffected cortical areas, which can develop and replace lost functions [85]. Visual feedback is most usual in VRBT [86], creating sensory illusions in patients during the active execution of movements. Thus, it can promote the reorganization of sensorimotor circuits, resulting in an improvement of postural balance and motor skills necessary to maintain dynamic balance [86]. VRBT has also been shown to be beneficial for the integration of vestibular and visual information through the vestibulo-ocular reflex, and consequently to improve balance [87]. VRBT favors the performance of standing activities, increasing muscular endurance in lower extremities and spine muscles that maintain the posture. It also involves the activation of muscle and joint proprioceptors, improving, therefore, somatosensory postural information [88].

Findings reported in this study are clinically relevant and provide the most appropriate VRBT dose for treating functional and dynamic balance in PwMS. One strength of our findings is that we provide the most optimal dose to obtain the largest improvement on functional and dynamic balance. One strength of our findings, with large interest for clinical practice, is that we report the most appropriate dose of VRBT to obtain the largest improvement for functional (at least 40 sessions, five sessions per week and 40–45 min per sessions), and for dynamic balance (between 8 and 19 weeks, twice per week and 20–30 min per session). Our findings provide support to the use of VRBT to recover balance in neurological diseases such as MS. The majority of the VR devices employed in the included studies are non-immersive, so the present results may be more valid for interventions based on non-immersive VR devices. As VRBT may be used both, in clinical settings and home, it may be also considered an excellent tool for tele-rehabilitation.

Assuming that the results reported in this meta-analysis are clinically relevant, some limitations must be considered however. Thus, the low number of participants per meta-analysis may reduce the accuracy of our findings, although studies involving neurological patients usually have small sample sizes. Furthermore, the small number of studies that assess some outcomes, such as balance confidence, fear of falling or postural control may also reduce the generalization of the findings. In addition, the medium risk of bias in the included studies, resulting from the impossibility of blinding participants and therapists, and assessors in sometimes, increases the selection risk, performance and detection biases. Other limitation is related to the risk of publication bias observed in some meta-analysis, and the impossibility of assessing this variable in some studies, which also reduces the generalization of the findings. Sensitivity analysis surpasses 20%, being another limitation that reduces the precision of our findings. Another limitation is related to the low quality evidence found in some meta-analysis, which can affect to the robustness of our findings. Finally, all the included studies conducted the assessment in the short-time, so it has not been possible to assess the effect of VRBT in the medium- and long-term.

## Conclusion

This review provides evidence supporting the effectiveness of VRBT to improve postural balance in PwMS. VRBT is better than UC, CT or RAGT to increase the functional balance, being able to exceed the MCID for BBS reported by scientific literature after VRBT. Therefore, VRBT can be considered an excellent strategy for functional balance rehabilitation in PwMS showing moderate disability. To increase functional balance VRBT would be applied during 40 sessions or more, five sessions per week and between 40 and 45 min. Regarding dynamic balance, VRBT shows a small effect, especially in PwMS with only minimal signs of disability. Our findings recommend that the more appropriated dose of VRBT protocols to improve dynamic balance would be between 8 and 19 sessions, with a duration of 20–30 min per session and twice per week. VRBT also improves different parameters related to postural control in EO and EC conditions. In addition, VRBT reduces the fear of falling compared with CT, increases balance confidence associated to ADLS. However, further RCTs studies using a larger sample size and a control of risk of bias are required in order to increase the generalizability of the present findings.

## Supplementary Information

**Additional** **file** **1. Supplementary Tables:** **Table** **S1.** Description of each Test Reported by Included Studies. **Table** **S2.** Subgroup Analysis According Disability Status (EDSS) of PwMS in Studies Included. **Table** **S3.** Subgroup Analysis According Total Number of Sessions. **Table** **S4.** Subgroup Analysis Number of Sessions per Week. **Table** **S5.** Subgroup Analysis According Duration of each Session in Minutes.

**Additional** **file** **2. Supplementary Figures**: **Figure** **S1.** Funnel Plot of the Effect of VRBT on Functional Balance. **Figure** **S2.** Funnel Plot of the Effect of VRBT on Dynamic Balance. **Figure** **S3.** Funnel Plot of the Effect of VRBT on Sway Area with Eyes Closed. **Figure** **S4.** Funnel Plot of the Effect of VRBT on Centre of Pressure Excursion with Eyes Open. **Figure** **S5.** Funnel Plot of the Effect of VRBT on Confidence of Balance. **Figure** **S6.** Funnel Plot of the Effect of VRBT on Fear of Falling. **Figure** **S7.** Funnel Plot of the Effect of VRBT on Gait Speed.

## Data Availability

Not applicable.

## Abbreviations

MS: Multiple sclerosis
CNS: Central nervous system
PwMS: Patients with multiple sclerosis
VRBT: Virtual reality-based therapy
niVRBT: Non-immersive virtual reality-based therapy
iVRBT: Immersive virtual reality-based therapy
ADLs: Activities of daily living
RCTs: Randomized controlled trials
CT: Conventional therapy
SMD: Standardized mean difference
95% CI: 95% Confidence interval
MCID: Minimally clinically important difference
EDDS: Expanded Disability Status Scale
UC: Usual care
RAGT: Robot assisted gait training
BBS: Berg Balance Scale
TUGT: Timed up & go-test
CoP: Center of pressure
ABC: Activities-specific balance confidence
FES-I: Falls Efficacy Scale (FES-1)
10MWT: 10 Meters’ walk test
25FWT: Timed 25-foot walk test
