# Supplementary material for: Virtual reality-based therapy improves balance and reduces fear of falling in patients with multiple sclerosis. a systematic review and meta-analysis of randomized controlled trials
Source: J Neuroeng Rehabil. 2023 Apr 11;20:42. doi: 10.1186/s12984-023-01174-z (PMC10088228; doi:10.1186/s12984-023-01174-z)
Supplement: Supplementary file 1 — Additional file 1. Supplementary Tables: Table S1. Description of each Test Reported by Included Studies. Table S2. Subgroup Analysis According Disability Status (EDSS) of PwMS in Studies Included. Table S3. Subgroup Analysis According Total Number of Sessions. Table S4. Subgroup Analysis Number of Sessions per Week. Table S5. Subgroup Analysis According Duration of each Session in Minutes. [file 12984_2023_1174_MOESM1_ESM.docx]

**Table S1.** Description of each Test Reported by Included Studies

| OUTCOME | TEST | DESCRIPTION |
| --- | --- | --- |
| FUNCTIONAL BALANCE | **Berg Balance Scale (BBS)** | The BBS is considered the gold standard for functional balance. It is a questionnaire comprised by tasks, ranging from standing up from a sitting position, to standing on one foot. Each task is scored from 0 (unable) to 4 (independent). The total score is 56 points, and large scores indicate poor functional balance.[1] |
| DYNAMIC BALANCE | **Timed Up & Go-Test (TUGT)** | The TUGT assesses the mobility skills to maintain the dynamic balance. It counts the time spent in which a person takes to rise from a chair, walk 3 meters, turn around, walk back to the chair, and sit down. The longer the time to perform the test, the patient has a worse dynamic balance and a greater risk of falls.[2] |
|  | **Four Square Step Test (4SST)** | The 4SST is a measure of dynamic balance. This test register the time spent to while the subject rapidly steps over low obstacles in clockwise and counterclockwise directions. Long time are related with poor dynamic balance.[3] |
| POSTURAL CONTROL (POSTUROGRAPHY) | **Sway Area** | Area in mm^2^ in which the ellipse encloses 95% of instantaneous positions of the center of pressure anterior, posterior, medial and lateral coordinates.[4] The smaller the sway area (in mm^2^), the better postural control. |
|  | **Center of Pressure Excursion** | Is defined as the absolute length of the center of pressure path movements thought testing period.[5] The smaller the center of pressure excursion, the better postural control. |
| CONFIDENCE OF BALANCE | **Activities-Specific Balance Confidence (ABC) scale** | The ABC is a scale composed by 16 items which assess the balance confidence level while performing different activities of daily living. The scores can range from 0 (poor balance confidence) to 100 (excellent balance confidence).[6] |
| FEAR OF FALLS | **Falls Efficacy Scale (FES-I)** | The FES-I is a patient self-reported questionnaire comprised by 16 activities of daily living to assess the level of concern relating to falls. The scores can range from 16 to 64 and the higher score, the more the fear of falling.[7] |
| WALKING  SPEED | **10 Meters’ Walk test (10MWT)** | The 10MWT is a reliable to assess the walking speed. Patients must walk on a flat, hard floor at their fastest speed for 10 meters. Higher scores indicate major walking speed.[8] |
|  | **Timed 25-Foot Walk Test (25FWT)** | The 25FWT is a measure to walking speed, in which a patient must walk a distance of 25 foot (7.6 meters approx.). Walking speed is high when the time spent is lower.[9] |

1. Berg KO, Wood-Dauphinee SL, Williams JI, Maki B. Measuring balance in the elderly: validation of an instrument. Can J Public Health. Switzerland; 1992;83 Suppl 2:S7-11.

2. Podsiadlo D, Richardson S. The timed “Up & Go”: a test of basic functional mobility for frail elderly persons. J Am Geriatr Soc. United States; 1991;39:142–8.

3. Dite W, Temple VA. A clinical test of stepping and change of direction to identify multiple falling older adults. Arch Phys Med Rehabil [Internet]. 2002;83:1566–71. Available from: https://linkinghub.elsevier.com/retrieve/pii/S0003999302002538

4. Brichetto G, Spallarossa P, de Carvalho MLL, Battaglia MA. The effect of Nintendo® Wii® on balance in people with multiple sclerosis: a pilot randomized control study. Mult Scler J [Internet]. 2013;19:1219–21. Available from: http://journals.sagepub.com/doi/10.1177/1352458512472747

5. Kalron A, Fonkatz I, Frid L, Baransi H, Achiron A. The effect of balance training on postural control in people with multiple sclerosis using the CAREN virtual reality system: a pilot randomized controlled trial. J Neuroeng Rehabil [Internet]. 2016;13:13. Available from: http://www.jneuroengrehab.com/content/13/1/13

6. Montilla-Ibanez A, Martinez-Amat A, Lomas-Vega R, Cruz-Diaz D, Torre-Cruz MJD la, Casuso-Perez R, et al. The Activities-specific Balance Confidence scale: reliability and validity in Spanish patients with vestibular disorders. Disabil Rehabil. England; 2017;39:697–703.

7. van Vliet R, Hoang P, Lord S, Gandevia S, Delbaere K. Falls Efficacy Scale-International: A Cross-Sectional Validation in People With Multiple Sclerosis. Arch Phys Med Rehabil [Internet]. 2013;94:883–9. Available from: https://linkinghub.elsevier.com/retrieve/pii/S0003999312012129

8. Steffen T, Seney M. Test-Retest Reliability and Minimal Detectable Change on Balance and Ambulation Tests, the 36-Item Short-Form Health Survey, and the Unified Parkinson Disease Rating Scale in People With Parkinsonism. Phys Ther [Internet]. 2008;88:733–46. Available from: https://academic.oup.com/ptj/article/88/6/733/2742317

9. Cutter GR. Development of a multiple sclerosis functional composite as a clinical trial outcome measure. Brain [Internet]. 1999;122:871–82. Available from: https://academic.oup.com/brain/article-lookup/doi/10.1093/brain/122.5.871

**Table S2.** Subgroup Analysis According Disability Status (EDSS) of PwMS in Studies Included

| Outcomes | Disability (EDSS) | Studies | N | Cohen’s SMD, 95% CI and *p*-value |
| --- | --- | --- | --- | --- |
| Functional balance | **Only minimal disability** | Ozkul (2020) | 39 | -0.02 (-0.76 to 0.72); *p* = 0.96 |
|  | **Minimal disability** | Not reported | - | Not reported |
|  | **Moderate disability** | Brichetto (2013), Ortiz-Gutiérrez (2013) | 83 | 1.91 (1.19 to 2.63); *p* < 0.001 |
|  | **Severe disability** | Calabrò (2017), Kalron (2016), Molhemi (2021), Peruzzi (2016), Yazgan (2020) | 191 | 0.69 (0.27 to 1.1); *p* = 0.001 |
|  | **Restricted ADL** |  |  | 0.99 (0.33 to 1.65); *p* = 0.003 |
| Dynamic balance | **Only minimal disability** | Ozkul (2020) | 39 | -0.51 (-1.29 to -0.28); *p* = 0.049 |
|  | **Minimal disability** | Not reported | - | Not reported |
|  | **Moderate disability** | Prosperini (2013) | 72 | -0.18 (-0.91 to 0.55); *p* = 0.62 |
|  | **Severe disability** | Calabrò (2017), Kalron (2016), Molhemi (2021), Peruzzi (2016), Yazgan (2020) |  | -0.27 (0.71 to 0.16); *p* = 0.22 |
|  | **Restricted ADL** | Not reported | - | Not reported |

Abbreviations: N = Number of participants; SMD = Standardized Mean Difference; 95% CI = 95% Confidence Interval.

**Table S3.** Subgroup Analysis According Total Number of Sessions

| Outcomes | Number of sessions | Studies | N | Cohen’s SMD, 95% CI and *p*-value |
| --- | --- | --- | --- | --- |
| Functional balance | 8-19 | Brichetto (2013), Kalron (2016), Khalil (2018), Lozano-Quilis (2014), Molhemi (2021), Munari (2020), Ozkul (2020), Peruzzi (2016), Yazgan (2020) | 297 | 0.55 (0.24 to 0.86); *p* = 0.001 |
|  | 20-39 | Eftekharsadat (2015), Tollar (2019) | 84 | 0.93 (0.33 to 1.52); *p* = 0.002 |
|  | >40 | Calabrò (2017), Ortiz-Gutiérrez (2013) | 87 | 1.9 (1.2 to 2.59); *p* < 0.001 |
| Dynamic balance | 8-19 | Kalron (2016), Khalil (2018), Lozano-Quilis (2014), Molhemi (2021), Nilsagard (2012), Ozkul (2020), Peruzzi (2016), Yazgan (2020) | 326 | -0.35 (-0.6 to -0.07); *p* = 0.012 |
|  | 20-39 | Eftekharsadat (2015), Maggio (2020) | 90 | -0.13 (-0.58 to 0.32); *p* = 0.58 |
|  | >40 | Calabrò (2017), Prosperini (2013) | 112 | -0.32 (-0.84 to 0.21); *p* = 0.24 |

Abbreviations: N = Number of participants; SMD = Standardized Mean Difference; 95% CI = 95% Confidence Interval

**Table S4.** Subgroup Analysis Number of Sessions per Week

| Outcomes | Number of sessions per week | Studies | N | Cohen’s SMD, 95% CI and p-value |
| --- | --- | --- | --- | --- |
| Functional balance | 1 | Not reported | - | Not reported |
|  | 2 | Eftekharsadat (2015), Kalron (2016), Khalil (2018), Munari (2020), Ozkul (2020), Yazgan (2020) | 252 | 0.41 (0.08 to 0.74); *p* = 0.016 |
|  | 3 | Brichetto (2013), Molhemi (2021), Peruzzi (2016) | 100 | 0.84 (0.32 to 1.36); *p* = 0.002 |
|  | 4 | Not reported | - | Not reported |
|  | 5 | Calabrò (2017), Tollar (2019) | 94 | 1.31 (0.76 to 1.86); *p* < 0.001 |
| Dynamic balance | 1 | - | - | Not reported |
|  | 2 | Eftekharsadat (2015), Kalron (2016), Khalil (2018), Nilsagard (2012), Ozkul (2020), Yazgan (2020) | 281 | -0.4 (-0.68 -0.11); *p* = 0.007 |
|  | 3 | Maggio (2020), Molhemi (2021), Peruzzi (2016) | 124 | -0.15 (-0.6–0.28); *p* = 0.49 |
|  | 4 | - | - | Not reported |
|  | 5 | Calabrò (2017), Prosperini (2013) | 112 | -0.12 (-0.58–0.32); *p* = 0.58 |

Abbreviations: N = Number of participants; SMD = Standardized Mean Difference; 95% CI = 95% Confidence Interval

**Table S5.** Subgroup Analysis According Duration of each Session in Minutes

| Outcomes | Minutes of Duration of each Session | Studies | N | Cohen’s SMD, 95% CI and p-value |
| --- | --- | --- | --- | --- |
| Functional balance | 20-30 | Eftekharsadat (2015), Kalron (2016), Molhemi (2021), Ortiz-Gutiérrez (2013), Ozkul (2020), Peruzzi (2016) | 223 | 0.69 ( 0.12 to 1.21); *p* = 0.016 |
|  | 40-45 | Calabrò (2017), Munari (2020) | 55 | 1.09 (0.02 to 2.15); *p* = 0.045 |
|  | 60 | Brichetto (2013), Lozano-Quilis (2014), Tóllar (2019), Yazgan (2020) | 158 | 0.77 (0.27 to 1.49); *p* = 0.005 |
| Dynamic balance | 20-30 | Eftekharsadat (2015), Kalron (2016), Molhemi (2021), Nilsagard (2012), Ozkul (2020), Peruzzi (2016), Prosperini (2013) | 328 | -0.36 (-0.62 to -0.1)*; p* = 0.01 |
|  | 40-45 | - | - | Not reported |
|  | 60 | Lozano-Quilis (2014), Maggio (2020), Yazgan (2020) | 128 | -0.2 (-0.62 to 0.22); *p =* 0.35 |

Abbreviations: N = Number of participants; SMD = Standardized Mean Difference; 95% CI = 95% Confidence Interval
