# Supplementary material for: Virtual reality-based therapy improves balance and reduces fear of falling in patients with multiple sclerosis. a systematic review and meta-analysis of randomized controlled trials
Source: J Neuroeng Rehabil. 2023 Apr 11;20:42. doi: 10.1186/s12984-023-01174-z (PMC10088228; doi:10.1186/s12984-023-01174-z)
Supplement: Supplementary file 2 — Additional file 2. Supplementary Figures: Figure S1. Funnel Plot of the Effect of VRBT on Functional Balance. Figure S2. Funnel Plot of the Effect of VRBT on Dynamic Balance. Figure S3. Funnel Plot of the Effect of VRBT on Sway Area with Eyes Closed. Figure S4. Funnel Plot of the Effect of VRBT on Centre of Pressure Excursion with Eyes Open. Figure S5. Funnel Plot of the Effect of VRBT on Confidence of Balance. Figure S6. Funnel Plot of the Effect of VRBT on Fear of Falling. Figure S7. Funnel Plot of the Effect of VRBT on Gait Speed. [file 12984_2023_1174_MOESM2_ESM.docx]

**Additional Figures**


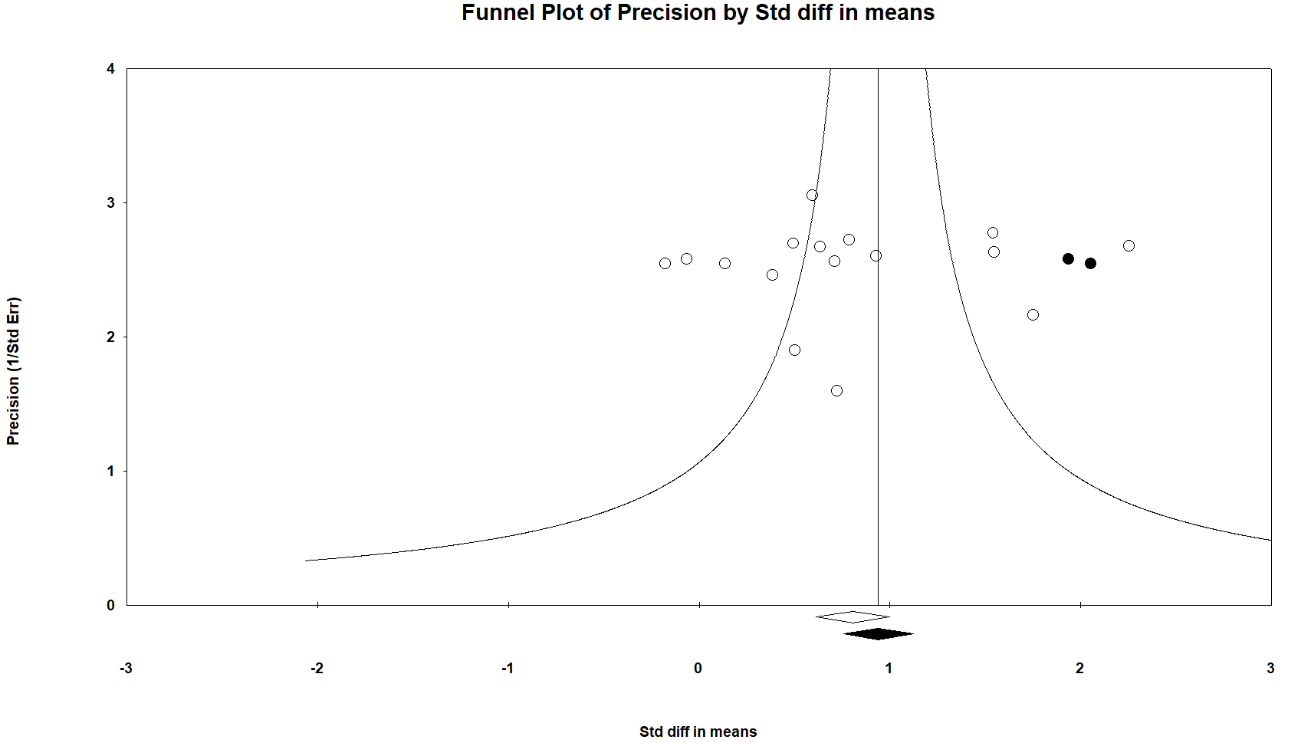


**Figure S1.** Funnel Plot of the Effect of VRBT on Functional Balance


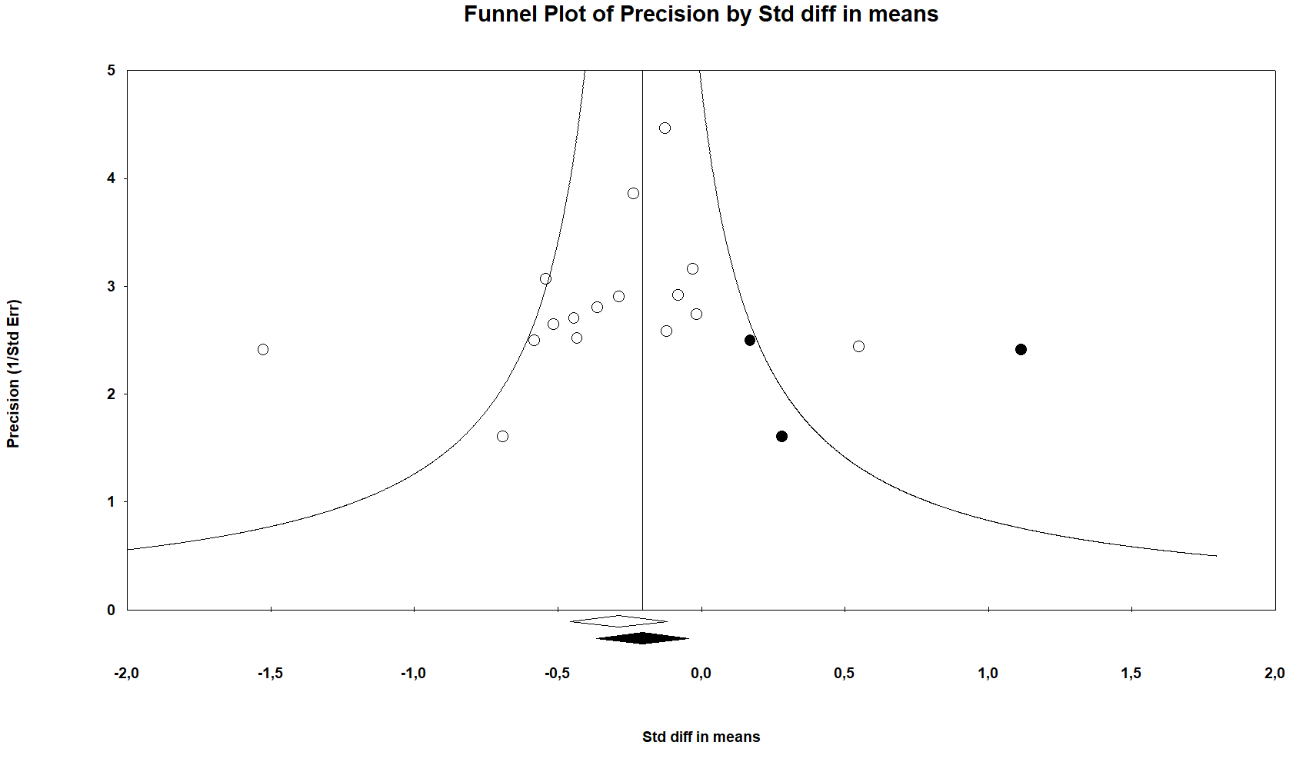


**Figure S2.** Funnel Plot of the Effect of VRBT on Dynamic Balance

**
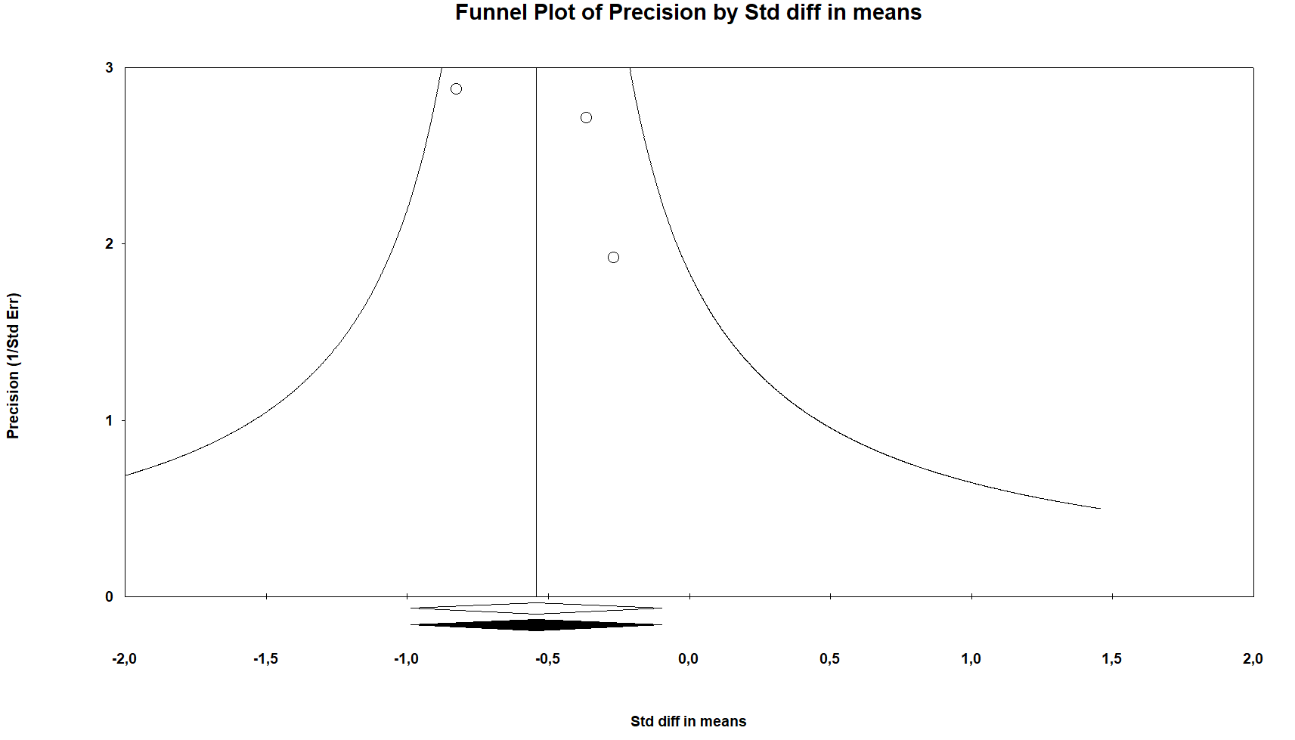
**

**Figure S3.** Funnel Plot of the Effect of VRBT on Sway Area with Eyes Closed


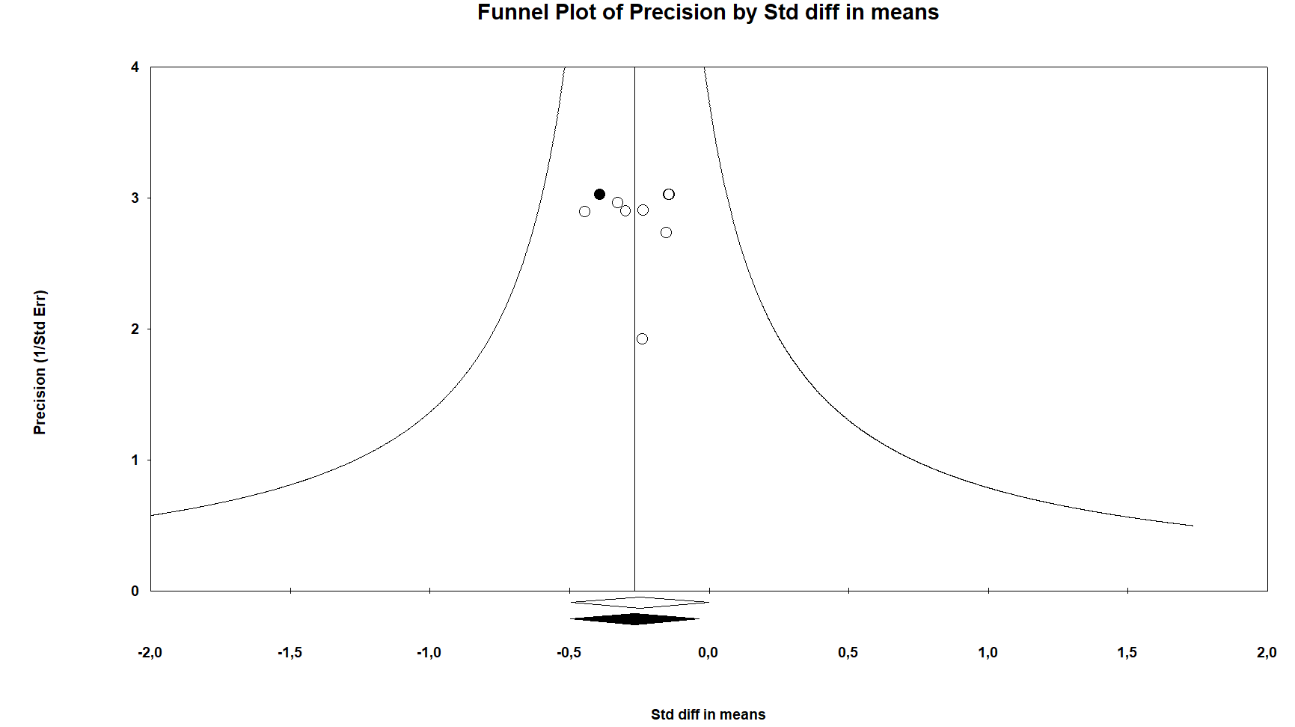


**Figure S4.** Funnel Plot of the Effect of VRBT on Centre of Pressure Excursion with Eyes Open


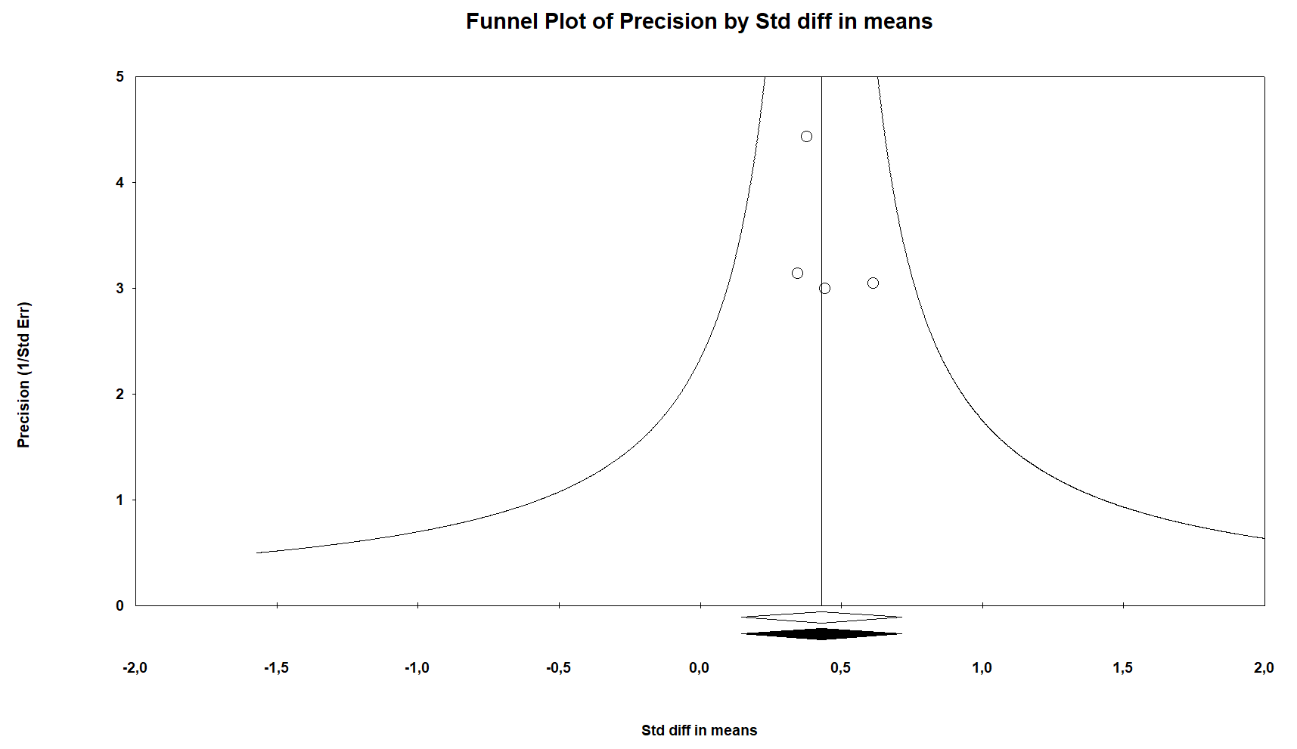


**Figure S5.** Funnel Plot of the Effect of VRBT on Confidence of Balance


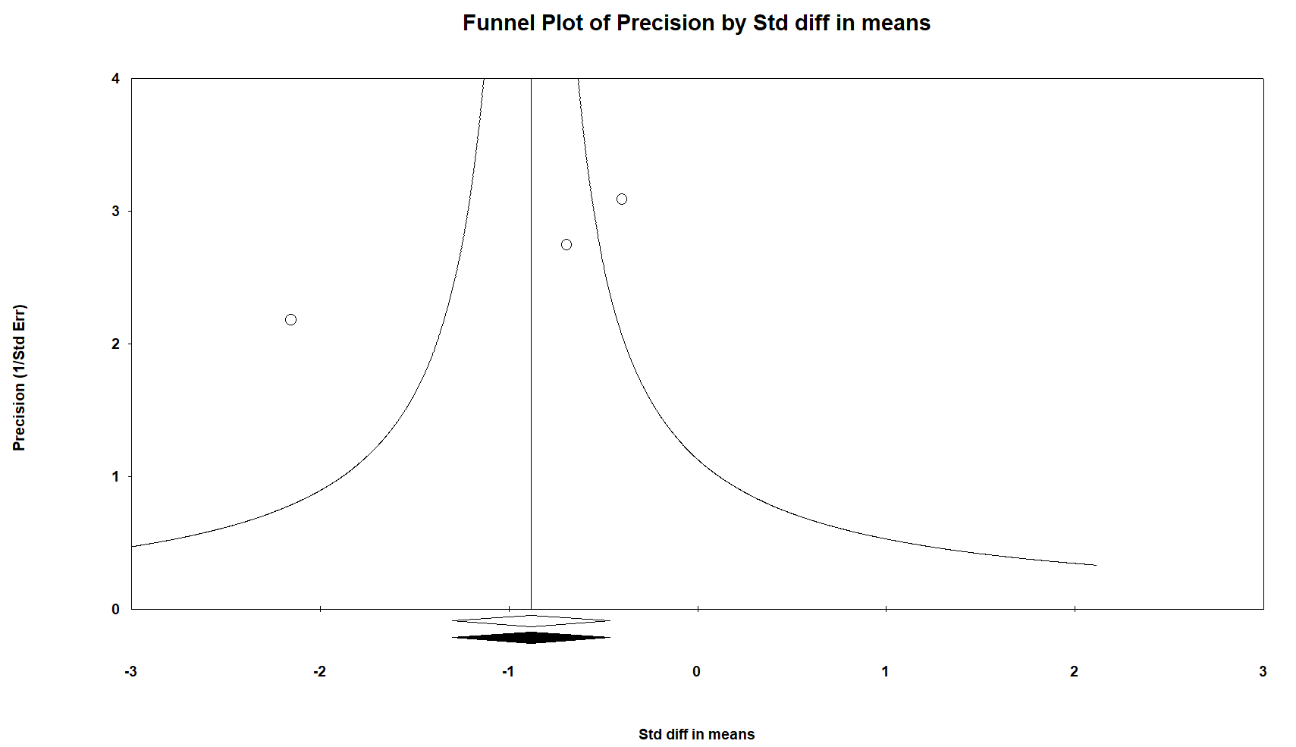


**Figure S6.** Funnel Plot of the Effect of VRBT on Fear of Falling

**Figure S7.** Funnel Plot of the Effect of VRBT on Gait Speed
